# Using nonlinear auto-regressive with exogenous input neural network (NNARX) in blood glucose prediction

**DOI:** 10.1186/s42234-024-00141-w

**Published:** 2024-04-17

**Authors:** Fayrouz Allam

**Affiliations:** Automatic control department, Tabin Institute For Metallurgical Studies, Iron and Steel street, Tabbin, Helwan, Cairo, Egypt

**Keywords:** Recurrent neural network, Nonlinear auto regressive model, Continuous glucose monitoring, Blood glucose prediction

## Abstract

**Background:**

Predicting of future blood glucose (BG) concentration is important for diabetes control. Many automatic BG monitoring or controlling systems use BG predictors. The accuracy of the prediction for long prediction time is a major factor affecting the performance of the control system. The predicted BG can be used for glycemia management in the form of early hypoglycemic/hyperglycemic alarms or adjusting insulin injections. Recent developments in continuous glucose monitoring (CGM) devices open new opportunities for glycemia management of diabetic patients. Many of those systems need prediction for long prediction horizons to avoid going through hypo or hyperglycemia.

**Methods:**

In this article a nonlinear autoregressive exogenous input neural network (NNARX) is proposed to predict the glucose concentration for longer prediction horizons (PHs) than that was obtained previously with an established recurrent neural network (RNN). The proposed NNARX is a modified version from our previously published RNN with different initialization and building technique but has the same architecture. The modification is based on starting with building nonlinear autoregressive exogenous input model using MATLAB and train it, then close the loop to get NNARX network.

**Results:**

The results of using the proposed NNARX indicate that the proposed NNARX is better in prediction and stability than unmodified RNN as PH becomes higher than 45 minutes.

**Conclusions:**

Modification in RNN building extends the ability of the prediction till 100 minutes. It performs statistically significant improvements in the FIT and RMSE values for 100 minutes prediction. It also decreases root mean squared error (RMSE) for both 45 and 60 minutes of prediction.

## Introduction

A chronic condition known as type-1 diabetes mellitus is defined by the pancreas’s failure to produce enough insulin. There are many useful tools on the market right now that can assist patients in regulating and monitoring their blood glucose (BG) levels. Continuous glucose monitoring (CGM) systems are used by the majority of the devices (Oviedo et al. 2017). Continuously checking the blood sugar level is known as monitoring, and automatically administering insulin or setting off alarms in response to the reading is known as controlling. It is well known that there is a delay between the administration of insulin and its effects (the appearance of maximum insulin value in the blood). The automatic control systems of BG prefer prediction of glucose due to this delay (Allam et al. 2013; Fernandez de Canetea et al. 2012). When using pumps, this prediction can be utilized to change insulin infusion rates or to set early hypoglycemic/hyperglycemic alarms. As a result, BG controlling is impacted by the BG prediction’s performance. The BG predictor’s prediction horizon should account for the time it takes for insulin to be absorbed and appear in the blood. The on-set time, or the rate at which insulin acts, and the peak time, or the moment when the effect is greatest, are used to define the delay (Diabetes Education Online n.d.). The kind of insulin determines all time delays. The rapid active insulin has a five-to-15-minute initiation of action and a one-to-two-hour peak impact. The beginning of action for regular insulin is between 0.5 and 1 hour, and the maximal effect is between 2 and 4 hours (Diabetes Education Online n.d.). Numerous studies have been conducted in this area to forecast future blood glucose levels and account for the various insulin action delay values. The challenge lies in obtaining predicted blood glucose levels that are accurate across extended time horizons in order to account for the time lags created by the various forms of insulin. The blood glucose (BG) predictor is utilized for both low and high glycemia alerts, as well as for predicting BG in a closed loop blood glucose (BG) controller. Based on the BG prediction, the control system determines the appropriate insulin dose to provide.

To anticipate the blood glucose level, many artificial intelligence (AI) algorithms are employed (Li et al. 2020). One of those well-liked AI methods, neural networks, was successful in BG prediction, particularly at short prediction horizons (Allam et al. 2011a; Allam et al. 2011b). Some researchers employ Artificial Neural Networks (ANN) in BG trend prediction (Tronstad and et al. 2019) to get better performance than BG level prediction. From CGM data, meal information, and insulin injection, other researchers (Zecchin et al. 2014a) employed RNN to predict BG. They forecast with an RMSE of 10.09 mg/dl for just 15 minutes. Because of the delayed impact of the injected insulin, BG management improves as prediction horizon (PH) increases. The feed forward neural network (FFNN) has been employed in the past, however it performs poorly in scenarios with long prediction horizons (Allam et al. 2013; Robertson et al. 2011; Ali et al. 2018). With the aid of partial parameter optimization, a novel amalgamation of the incremental learning and echo state network (ESN) techniques is constructed in (Li et al. 2020). The proposed approaches yield outstanding prediction performance at low PH, according to experimental results reported in (Zecchin et al. 2014b), and their performance declined as PH grew. A jump NN prediction technique (horizon 30 minutes) using both consumed carbohydrate information and previous CGM data was proposed in (Zecchin et al. 2014b). Using CGM data of past glucose concentration values, which are created by the sliding window technique, an ANN prediction model with time-domain properties was proposed in (Alfian et al. 2020a) (Alfian et al. 2020b). For PH equal to 15, 30, 45, and 60 minutes, the model in (Alfian et al. 2020a) has been used. Other ANN algorithms (Bertachi et al. 2018) combine input data for meals, insulin, and physical activity in addition to CGM data.

Non-linear auto-regressive exogenous input is one AI approach (NARX). A recurrent neural design frequently utilized for input-output modelling of nonlinear systems is the NARX network (Ardalani-Farsa and Zolfaghari 2010; Miky et al. 2021; Heidari et al. 2020). It is well known that NARX nets perform similarly to recurrent neural networks (RNNs) in terms of modelling (Sum et al. 1999) and do not suffer from RNNs’ poor performance in terms of long-term prediction (Ruiz et al. 2016). Additionally, it is demonstrated that NARX is a potent Turing machine when only a small number of nodes and taps are used (Siegelmann et al. 1997). Fast convergence and strong aptitude for problems with long-term dependencies are the key benefits of NARX nets (Lin et al. 1996).

Earlier in (Tian et al. 2018), NNARX and recurrent neural network (RNN) were used to enhance streamflow estimation. Combining these two factors enhanced forecast accuracy. The NNARX network is employed in this study to improve RNN’s BG prediction ability for higher PH values. We will construct the NNARX network by starting with the NNARX open loop network, closing the loop to convert it to the NNARX closed loop neural network, and evaluating the performance of BG prediction. The aim of this study is to enhance the performance of RNN in long time prediction horizon. The following outline describes the structure of this paper: in section 2, the size, shape, and preparation of the dataset are discussed. In this section also, the process of constructing and training NNARX is outlined for the reader. In section 3, the testing and evaluation metrices are introduced. The results are presented in the fourth section. Discussions of results and the conclusions are presented in sections 5 and 6 respectively.

## Methods

The proposed technique is evaluated using glucose measurements from the Diabetes Research in Children Network (DirecNet) Website (Diabetes Research in Children Network (DirecNet) 2009), which generates continuous glucose data for six distinct studies. We utilized 4916 samples from 9 subjects in one of the DirecNet studies. The participants ranged in age from three to 18 years and had been identified as having type 1 diabetes for over a year and had been using insulin pump. The subjects were provided with the Guardian RT CGM system for home usage. The duration of glucose measurements for each patient was an average of two days, and each day consisted of 288 samples. There are 4916 samples in our data collection overall after excluding the glucose values that have large gap during the measurements. One subset of this data (first 500 samples) is used for testing and validating the model, and the other subset (4416 samples) is used for training. These subsets were not stratified, we use data samples for training as much as we can. Prior to training and evaluating the neural networks, the data underwent smoothing through the implementation of the smooth () function in MATLAB, which employs the moving average technique. It was implemented with span 11. The time lag between the projected glucose and observed glucose values is decreased by using smoothed versions of the CGM data (Gani et al. 2010).

**Fig. 1:**
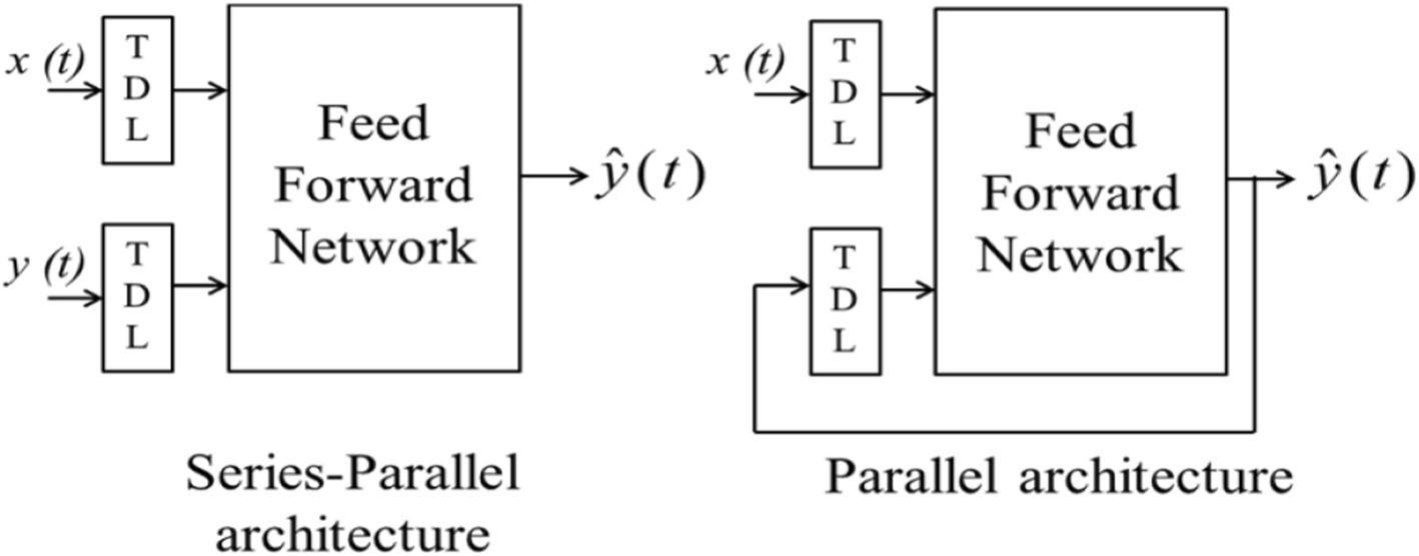
Architecture of **N**NARX

As seen in Fig. 1, the NNARX model has two alternative architectures: an open-loop, series-parallel design, and a closed-loop, parallel architecture, both of which are determined by Eqs. (1) and (2), respectively (Gani et al. 2010):

1 $$\left(t+1\right)=F\left(\begin{array}{c}y(t),y\left(t-1\right),\cdots, y\left(t-{n}_y\right),x\left(t+1\right),\\ x(t),x\left(t-1\right),\cdots, x\left(t-{n}_x\right)\end{array}\right)$$

2 $$\hat{y}\left(t+1\right)=F\left(\begin{array}{c}\hat{y}(t),\hat{y}\left(t-1\right),\cdots, \hat{y}\left(t-{n}_y\right),x\left(t+1\right),\\ x(t),x\left(t-1\right),\cdots, x\left(t-{n}_x\right)\end{array}\right)$$

**Fig. 2:**
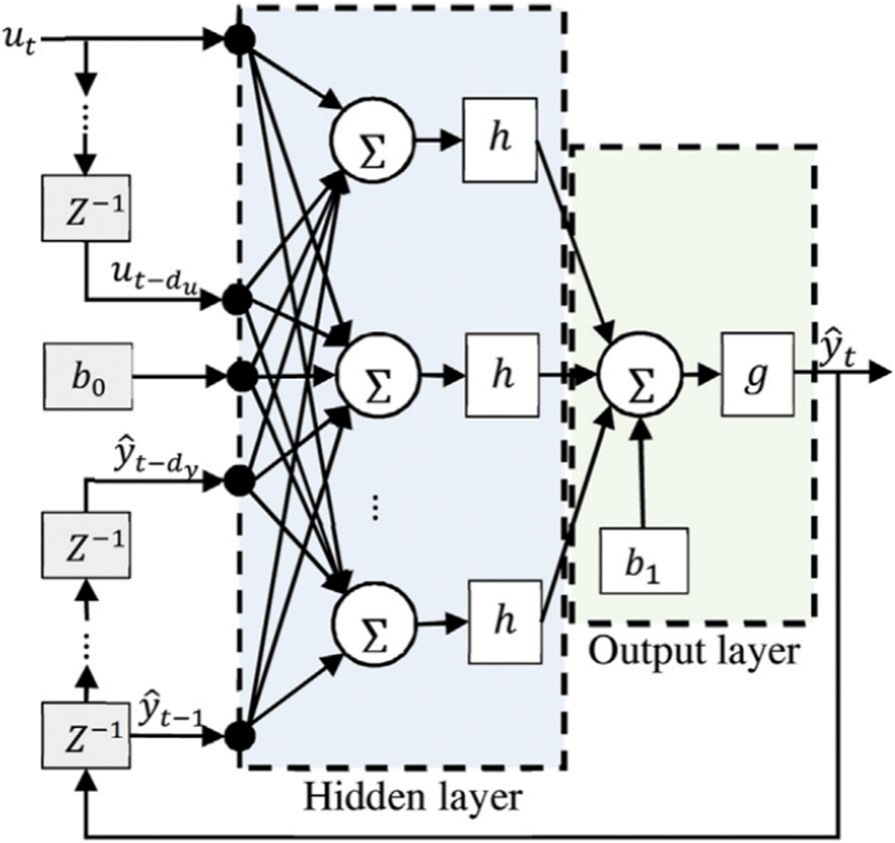
NNARX network architecture (Lin et al. 1996)

Equation 1 illustrates how the future value of the time series *y*^^^(*t* + 1) in the series-parallel architecture is predicted from the current value of *x(t)*, previous inputs *x(t-1)* to *x(t-n*_*x*_*),* and the true previous values of the time series *y(t)*. In the training phase, this architecture is utilized. In the prediction phase, parallel architecture is utilized (Boussaada et al. 2018). The prediction in the parallel architecture is done using the current value *x(t)*, previous inputs *x(t-1)* to x*(t-n*_*x*_*)*, and previously predicted values of the time series *y(t)*. For multi-step-ahead prediction, the NNARX neural network is transformed into a parallel design (Ferreira et al. 2012; Buitrago and Asfour 2017). A more thorough representation of the NNARX net architecture for prediction is shown in Fig. 2 (Lin et al. 1996). The long-term dependencies between a model’s output and its prior values, as well as its past and present values of an exogenous input, are performed via delay taps in the NNARX input. The nonlinear aspect of the process is made up of the hidden layers, which extract some information from the inputs and outputs.

### The steps of building and training NNARX are

**Fig. 3:**
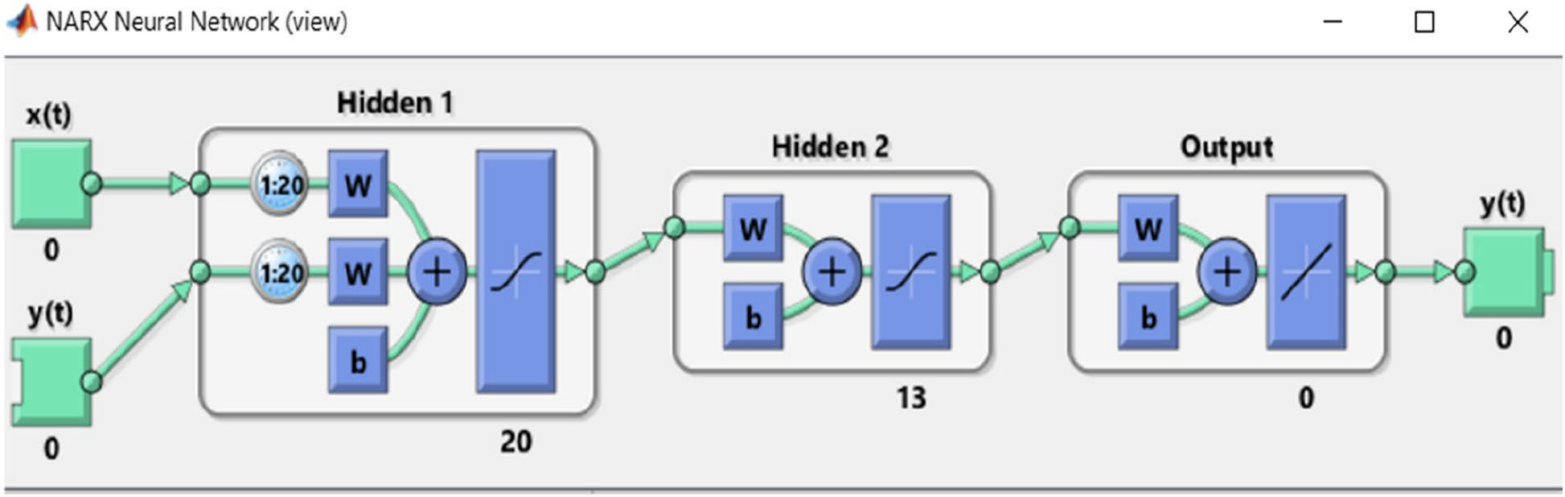
The proposed open loop NNARX

1. Create a two-hidden-layer NNARX model that contains 20 and 13 neurons respectively. Glucose concentration readings are used as all inputs. The architecture of the open loop NNARX is depicted in Fig. 3.
2. Train the open loop NNARX to predict after 20 samples (100 minutes). the training is performed by applying 40 samples each time,20 samples as inputs and 20 samples as output. This will happen till get the better prediction performance.

**Fig. 4:**
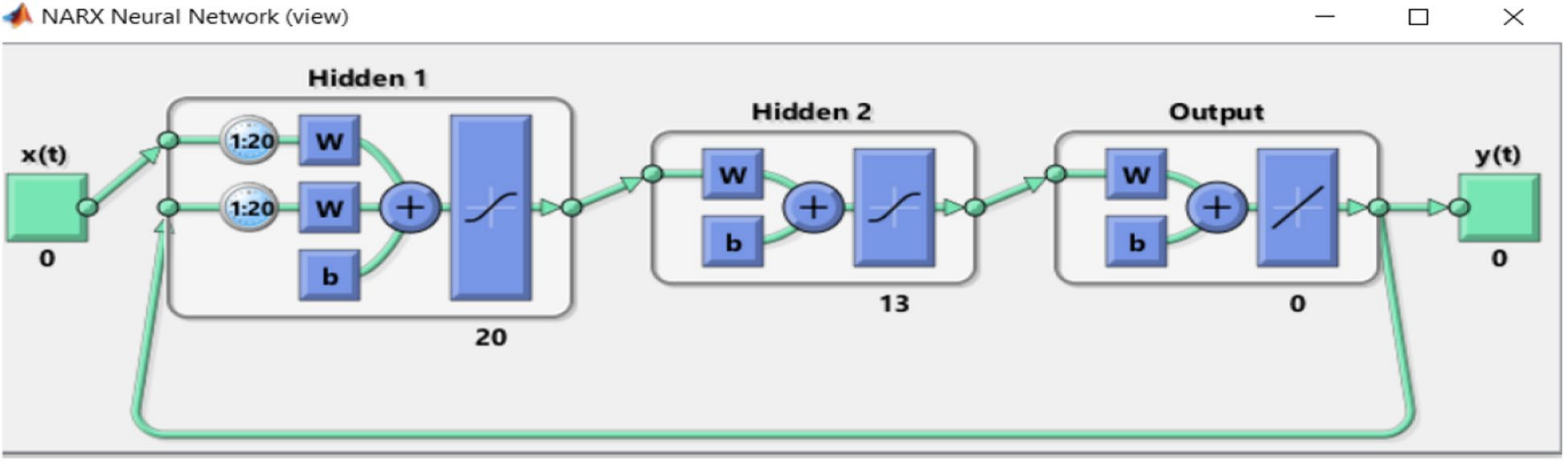
The proposed closed loop NNARX

Close the loop as shown in Fig. 4.

Apply the first 20 glucose values to the closed network and iteratively obtain the multi-step anticipated output. With correct performance, we can obtain the anticipated glucose after 5 to 100 minutes.

## Experimental

### Assessment metrics

Numerous performance indicators, including root mean squared error (RMSE), normalized prediction Error (NPE), and FIT are used to evaluate the performance of the proposed NNARX network.

The FIT metric represents the percentage of the data variation that can be accounted for by the model. A FIT of 0% indicates that its performance would be equivalent to predicting the mean measurement value. A perfect FIT corresponds to 100% explanation of the data variation. FIT metric is differed from R-squared which is a goodness-of-fit measure for linear regression models**.**

The following formulations correspond to these measures.

3 $$\boldsymbol{RMSE}=\sqrt{\frac{\sum_{\boldsymbol{i}=\textbf{1}}^{\boldsymbol{N}}\Big(\boldsymbol{Gi}-\hat{\boldsymbol{Gi}\Big)}\textbf{2}}{\boldsymbol{N}}}$$

4 $$\textbf{FIT}=\left(\textbf{1}-\frac{\left\Vert \boldsymbol{G}\left(\boldsymbol{t}\right)-{\boldsymbol{G}}^{\hat{\phantom{0}}}\left(\boldsymbol{t}\right)\right\Vert }{\left\Vert \boldsymbol{G}\left(\boldsymbol{t}\right)-{\boldsymbol{G}}^{-}\left(\boldsymbol{t}\right)\right\Vert}\right)\ast \textbf{100}$$

5 $$\boldsymbol{NPE}=\sqrt{\frac{\sum_{\boldsymbol{i}=\textbf{1}}^{\boldsymbol{N}}{\left(\boldsymbol{G}\left(\boldsymbol{i}\right)-\hat{\boldsymbol{G}\left(\boldsymbol{i}\right)}\right)}^{\textbf{2}}}{\sum_{\boldsymbol{i}=\textbf{1}}^{\boldsymbol{N}}{\boldsymbol{G}}^{\textbf{2}}\left(\boldsymbol{i}\right)}}\ast \textbf{100}$$

${\boldsymbol{G}}_{\boldsymbol{i}}^{\hat{\phantom{0}} }$ is the estimated glucose value for sample *i*, N is the total number of samples, *G*_*i*_ is the glucose reading for sample *i* and ${\boldsymbol{G}}_{\boldsymbol{i}}^{-}$ is the average of all glucose readings. The Clarke error grid approach (Clarke’s EGA) (Clarke 2005) was also utilized to evaluate the performance of the prediction algorithm with a clinically acceptable metric in addition to the three metrics mentioned above. Each sensor reading and its accompanying anticipated glucose concentration are mapped by Clarke’s EGA into five zones, A–E, with various degrees of accuracy and imprecision in glucose estimation. As a result, zone A stands for results that are accurate, zone B for results that are acceptable, zone C for overcorrections that may result in a poor clinical outcome, zone D for results that may indicate a potentially dangerous failure to detect hypoglycemia or hyperglycemia, and zone E for results that may cause treatment of hypoglycemia to be confused with treatment of hyperglycemia and vice versa.

The testing data consists of 500 samples obtained from type-1 diabetic patients who were under control. These samples encompass a broad spectrum of glucose readings, ranging from low values of 3.5 mmol/L to controlled values of 15.5 mmol/L. For various PH levels, the NNARX network’s prediction performance is assessed.

## Results

**Fig. 5:**
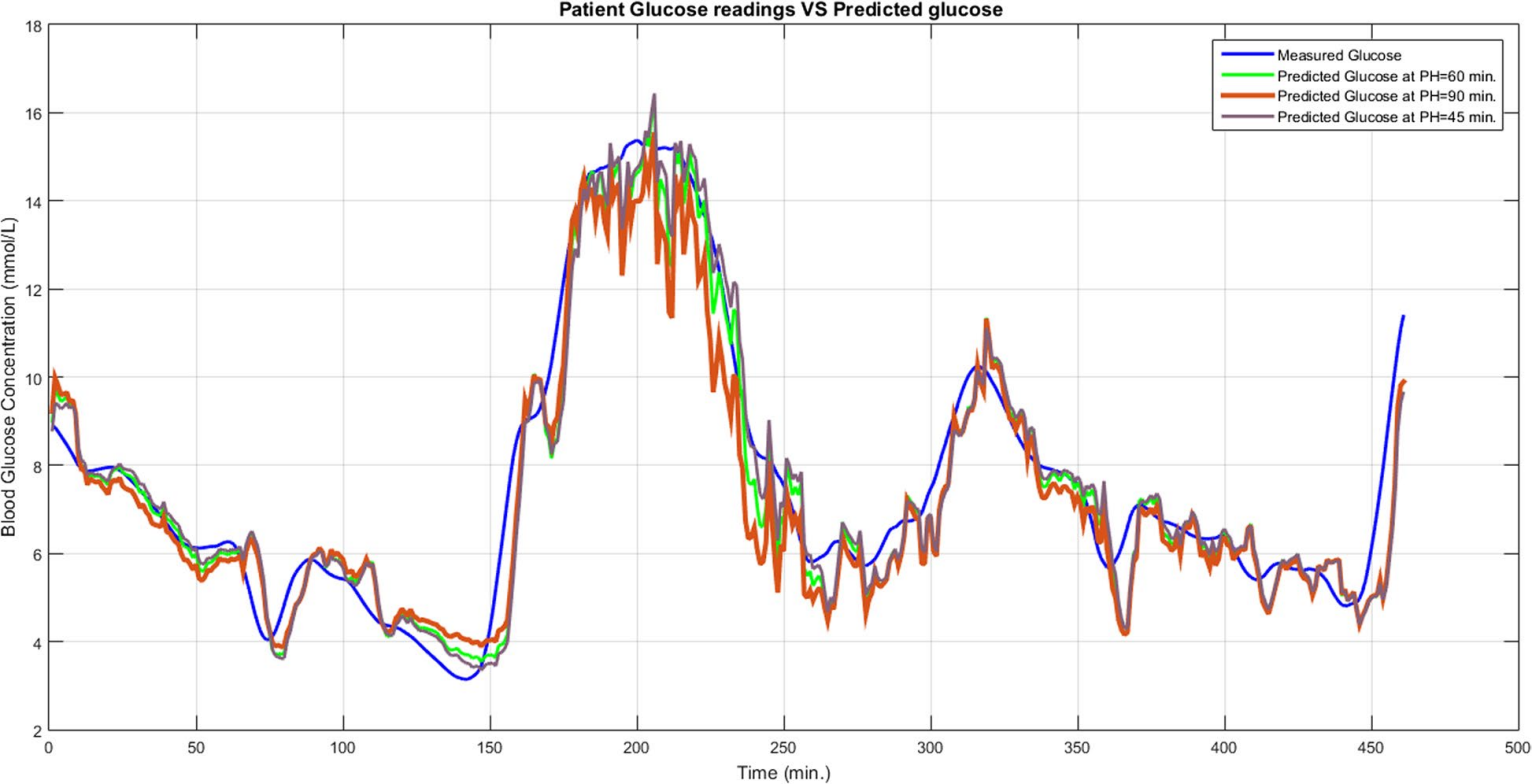
Patient glucose readings Versus Predicted glucose at PH = 45 min., 60 min., and 90 m

**Fig. 6:**
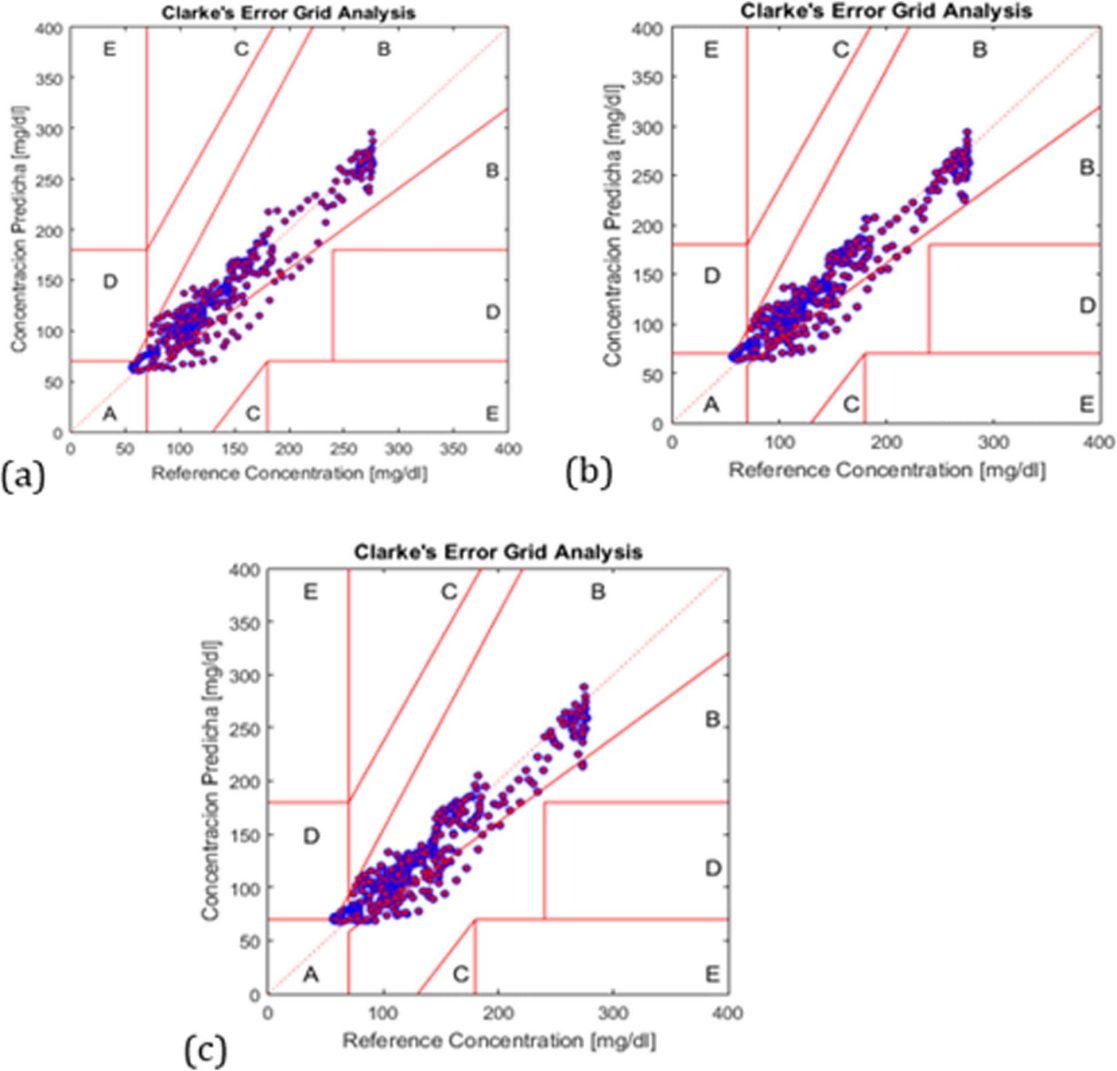
Clarck’s EGA for proposed NNARX at PH=: (**a**)45 min., (**b**) 60 min., (**c**) 90 min

The blood glucose levels predicted by our suggested NNARX for various PH values are shown in Fig. 5. The evaluation metrics calculated for the suggested NNARX are displayed in Table 1. The plot of the clinical evaluation of the predicted glucose values using Clarck’s EGA is shown in Fig.6. it shows that the predicted glucose values are located in the acceptable ranges (A, B). The statistical analysis of the data presented in Table 1 involves the utilization of a one-sample t-test to determine the two-tailed *P* values for PH = 90 and 100 minutes. P values are employed to demonstrate that the proposed NNARX predicts 90 and 100 minutes with statistically significant enhancement over other predicted values. RMSE has a P value of 0.007 at 100 minutes, whereas FIT has P values of 0.03 at 90 minutes, and 0.0082 at 100 minutes. All these values are below 0.05, indicating that the RMSE and FIT values are statistically significant. Consequently, there is a statistically significant improvement in the prediction accuracy at high PH values, which leads to an enhancement in the control of the insulin infusion devices.

**Table 1:** Evaluation Metrices for the prediction of the proposed NNARX

PH (min.)	NPE (%)	RMSE (mmol/L)	FIT (%)
15	14.1713	1.18	79.2484
30	11.6805	0.9117	82.9889
45	10.5562	0.7446	84.6989
60	10.6028	0.7512	84.0512
90	12.3604	1.0209	77.9620*
100	13.3	1.34*	75.2006*

*indicates a value that is a statistically significant *P* < 0.05

**Fig. 7:**
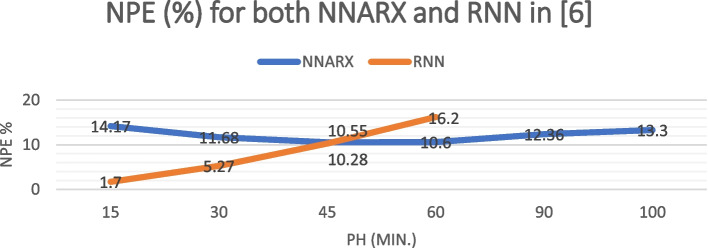
NPE for both NNARX and RNN in (Allam et al. 2011a) for different PH values

**Fig. 8:**
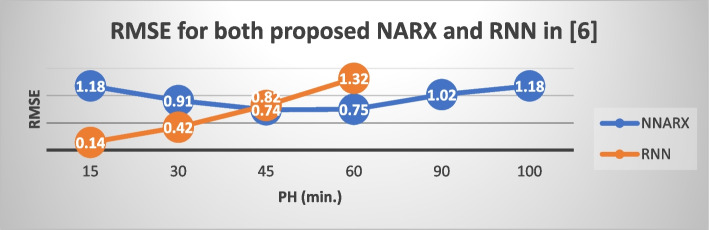
RMSE for both NNARX and RNN in (Allam et al. 2011a) for different PH values

We will now compare the NNARX prediction performance metrics with those previously defined in (Allam et al. 2011a) to determine how changes in the network’s construction and training will impact prediction outcomes, particularly over extended prediction horizons. Table 2 shows the comparison. The RNN in (Allam et al. 2011a) has the same architecture (20–13-1), the same training and testing data. According to (Allam et al. 2011a), their RNN could only provide acceptable prediction performance for a duration of 60 minutes. Table 2 therefore does not include evaluation metrics for RNN at the 90 and 100-minutes prediction horizon. The standard deviation values presented in Table 2 are computed for each model performance metric across the entire range of PH values. Figs. 7 and 8 show the NPE and RMSE for both NNARX and RNN in (Allam et al. 2011a) for different PH values. Both network findings had cross values around a 45-minute prediction, as seen in Figs. 7 and 8, Combining the two Figs. 7 and 8 is not suggested due to the disparity in range between RMSE and NPE%.

**Table 2:** Comparison of evaluation Metrices for the proposed NNARX and RNN in (Allam et al. 2011a)

PH (min.)	NPE (%)		RMSE (mmol/L)		FIT (%)	
	Proposed NNARX	RNN in (Allam et al. 2011a)	Proposed NNARX	RNN in (Allam et al. 2011a)	Proposed NNARX	RNN in (Allam et al. 2011a)
15	14.1713	1.7	1.1890	0.14	79.2484	95.33
30	11.6805	5.27	0.9117	0.42	82.9889	85.83
45	10.5562	10.28	0.7446	0.82	84.6989	72.3
60	10.6028	16.2	0.7512	1.32	84.0512	56.61
90	12.3604	–	1.0209	–	77.9620	–
100	13.3	–	1.1890	–	75.2006	–
Standard deviation	1.330464	6.29	0.180432	0.51	2.530228	17.01

The Clarke’s EGA (Clarke 2005) for the proposed NARX and RNN (Allam et al. 2011a) to clinically evaluate the predicted output for various PH values is displayed in Table 3. It demonstrates how the clinical evaluation for the 15 minute prediction is excellent (all samples are situated in zone A), and how the prediction using RNN degraded to get 1.95% of samples in zone D for the 60 minute prediction. In NNARX, this never occurs; all samples remain in zones A and B up until the prediction of 100 minutes.

**Table 3:** The Clarke’s EGA for Output of NNARX and RNN (Allam et al. 2011a) at Different prdeiction horizons

PH	% of samples in zone A		% of samples in zone B		% of samples in zone C		% of samples in zone D		%of samples in zone E	
	NNARX	RNN	NNARX	RNN	NARX	RNN	NARX	RNN	NARX	RNN
15	83.94	100	16.05	0	0	0	0	0	0	0
30	88.5	98.7	11.47	1.3	0	0	0	0	0	0
45	90.8	91.6	9.2	8.4	0	0	0	0	0	0
60	90.02	78.8	9.98	19.3	0	0	0	1.9	0	0
90	85.46	–	11.71	–	0	–	0	–	0	–
100	83.73	–	13.44	–	0	–	0.43	–	0	–

We can now compare our findings to those of other models, like those described in (Alfian et al. 2020a; Bertachi et al. 2018; Martinsson et al. n.d.; Allam 2021), that employ neural networks to predict blood glucose levels for a range of PH values. Other than glucose measurements, each of those neural networks has a unique training procedure and a wide range of input parameters. To demonstrate how the performance is good when the PH value increases, a comparison for 45 minutes and an hour is made, as shown in the Table 4. In (Alfian et al. 2020a; Allam 2021), neural network models were evaluated for prediction horizons 15,30,45, and 60 minutes. In (Bertachi et al. 2018; Martinsson et al. n.d.), the researchers evaluated their models to predict for two points only at 30 and 60 minutes.

**Table 4:** Comparison of RMSE values in (mmol/L) with other published neural network prediction models

PH (min.)	Our NNARX	Model proposed in (Alfian et al. 2020a)	Model proposed in (Martinsson et al. n.d.)	Model proposed in (Bertachi et al. 2018)	Model proposed in (Allam 2021)
45	0.7446	0.59	–	–	0.75
60	0.7512	0.85	1.74	1.76	1.19

Table 4 demonstrates how well our model compares to existing NN and CGM-based algorithms (Alfian et al. 2020a; Bertachi et al. 2018; Martinsson et al. n.d.; Allam 2021).

## Discussions

All performance indices utilizing the proposed NNARX have a lower standard deviation than those using the RNN in (Allam et al. 2011a), as demonstrated in Table 2. It means that, performance indices through different PH values are centered around their mean values. This also implies that the NNARX network’s performance is consistent over the range of PH values. While RNN networks perform well for predictions at low PH values, their performance degrades as PH increases (as shown in figs. 7 and 8). Comparing performance with other models (shown in Table 4) indicates that our NNARX can forecast for up to 60 minutes with an RMSE that is lower than that of other networks. This study is limited in prediction to the maximum number of inputs of the designed network.

## Conclusions

Patients with limited medical knowledge can benefit from using only prior glucose readings as inputs to neural networks that can accurately predict future results. From all the prior findings, modifying RNN construction (NNARX) and training open loop networks as opposed to closed loop networks improves prediction performance. For 45 minutes of prediction, the RMSE of prediction is reduced from 0.82 to 0.74 mmol/L, and for 60 minutes, it is reduced from 1.3 to 0.75 mmol/L. A change in RNN construction and training increases its capacity for prediction to 100 minutes with RMSE = 1.19 mmol/L. As a result, we can conclude that NNARX has good clinical and numerical performance in BG prediction. Our NNARX performs better across the full range of PH values from 15 to 100 minutes. In high PH values, our NNARX outperforms RNN, while it falls short in low PH values. Consequently, the NNARX network can be employed to predict long-term blood glucose levels; when this predictor is integrated with an insulin pump in a closed loop control system, BG level management is enhanced. The maximum achievable PH value is limited with the number of network inputs. It performs statistically significant improvement in 100 minutes prediction. As the number of inputs increases, NNARX can predict for longer prediction horizons.

## Data Availability

The datasets analyzed during the current study are available from the corresponding author on reasonable request.

## Abbreviations

BG: Blood Glucose
CGM: Continuous Glucose Monitoring
NNARX: Nonlinear Autoregressive Exogenous Input Neural Network
RNN: Recurrent Neural Network
ANN: Artificial Neural Network
PH: Prediction Horizon
FFNN: Feed Forward Neural Network
NPE: Normalized Prediction Error
RMSE: Root Mean Squared Error
CGA: Clarck’s Error Grid Analysis
